# Long-term changes in body weight and physical activity in relation to all-cause and cardiovascular mortality: the HUNT study

**DOI:** 10.1186/s12966-019-0809-2

**Published:** 2019-05-20

**Authors:** Anne Lovise Nordstoga, Ekaterina Zotcheva, Ellen Rabben Svedahl, Tom I. L. Nilsen, Eivind Schjelderup Skarpsno

**Affiliations:** 1 Department of Public Health and Nursing, Faculty of Medicine and Health Sciences, Norwegian University of Science and Technology, Håkon Jarls gate, 11, 7030 Trondheim, Norway; 2 Clinic of Anaesthesia and Intensive Care, St Olavs Hospital, Trondheim University Hospital, Trondheim, Norway; 3 Department of Neurology and Clinical Neurophysiology, St. Olavs Hospital, Trondheim, Norway

**Keywords:** Mortality, All-cause mortality, Cardiovascular mortality, Physical activity, Body weight, Weight gain, HUNT study

## Abstract

**Background:**

Most previous studies have relied on single measurements of body weight and physical activity and have not considered the interplay between long-term changes in body weight and physical activity in relation to mortality. The aim of the current study was therefore to examine the joint effect of changes in body weight and leisure-time physical activity over a period of ~ 10 years on all-cause and cardiovascular mortality.

**Methods:**

The study population comprised 34,257 individuals who participated in the first (1984–86) and second (1995–97) waves of the HUNT Study, and were followed up through the Norwegian Cause of Death Registry until December 31st, 2013. We used Cox regression to estimate hazard ratios (HR) with 95% confidence intervals (CI) of death associated with changes in body weight and leisure-time physical activity.

**Results:**

Compared to the reference group with stable weight who were long-term physically active, people who gained ≥5% of their weight had a HR for all-cause mortality of 1.54 (95% CI: 1.28–1.85) if they were long-term physically inactive; a HR of 1.23 (1.09–1.40) if they became physically active, and a HR of 1.00 (95% CI 0.94–1.06) if they were long-term physically active. The corresponding HRs for cardiovascular mortality were 1.57 (95% CI 1.17–2.12), 1.28 (95% CI 1.04–1.58) and 1.06 (95% CI 0.96–1.16), respectively. Long-term physical inactivity was associated with increased all-cause (HR 1.29; 95% CI 1.08–1.53) and cardiovascular (HR 1.37; 95% CI 1.05–1.79) mortality among those who were weight stable.

**Conclusions:**

The risk of all-cause and cardiovascular mortality is particularly evident among people who gain weight while remaining inactive during a ~ 10 year period. However, participants who remained physically active had the lowest risk of premature mortality, regardless of maintenance or increase in weight. These findings suggest that there is an interplay between long-term changes in body weight and physical activity that should receive particular attention in the prevention of premature mortality.

**Electronic supplementary material:**

The online version of this article (10.1186/s12966-019-0809-2) contains supplementary material, which is available to authorized users.

## Background

Weight gain has repeatedly been associated with an increased all-cause and cardiovascular mortality [1, 2]. However, favorable effects of weight gain on mortality have also been reported [3]. The explanation behind these inconsistent findings remains unknown, but may be partially related to the positive influence of leisure-time physical activity on mortality [4, 5]. Previous studies have shown that increasing the level of physical activity may reduce both all-cause and cardiovascular mortality considerably [6, 7].

Increasing evidence points towards a joint effect of body weight and physical activity on mortality, suggesting that physical activity can compensate the negative effect of excessive body weight on mortality [8–10]. Results from a large study showed that mortality in overweight and obese individuals was reduced among all physical activity groups when compared to inactive participants [10]. Another study showed that higher levels of physical activity attenuated some of the increased mortality among obese persons over a ten-year follow-up [11], but that the increased risk was not entirely eliminated. However, most previous studies have relied on single measurements of weight status and physical activity and have not considered the influence of long-term lifestyle changes on mortality. Since leisure-time physical activity and body weight may fluctuate throughout an individual’s life course, the effect on mortality may be better reflected by assessing the long-term changes in these factors. One large cohort study found that, irrespective of change or stability in BMI, men who became less fit had higher all-cause and cardiovascular mortality [12]. Although cardiorespiratory fitness is a good measure of physical capacity, it has a strong genetic component [13] and may be less modifiable than leisure-time physical activity habits. Thus, increased knowledge about the potential interplay between concurrent changes in body weight and leisure-time physical activity are necessary to guide public health recommendations aimed at preventing premature mortality.

The aim of this large population-based cohort study was therefore to prospectively investigate the joint association of concurrent changes in body weight and leisure-time physical activity with all-cause and cardiovascular mortality.

## Methods

### Participants

The HUNT Study is a population-based study performed in Nord-Trøndelag County, Norway [14]. All inhabitants aged 20 years and older were invited to participate in several consecutive surveys. The participation rate was 89% (*n* = 77,216) at HUNT1 (1984–86), and 66% (*n* = 65,237) at HUNT2 (1995–97). For the purpose of the present study, we included 45,925 persons who participated in both waves. The participants attended a clinical examination with measures of body weight and height, and filled in comprehensive questionnaires on lifestyle and health related factors, returned by mail subsequently. We excluded persons with incomplete information on weight (*n* = 351), and/or leisure-time physical activity (*n* = 11,317). Thus, the prospective analyses were based on information from 17,700 women and 16,557 men.

### Outcome

Participants in the HUNT Study were linked with the Norwegian Cause of Death Registry at Statistics Norway to prospectively assess incident cases of death in the cohort. Death from cardiovascular disease was classified according to the International Classification of Diseases (ICD-9: 390–459, and ICD-10: I00-I99). Each participant contributed person-years from the date of participation in HUNT2 until the date of death, emigration, or end of follow-up at December 31st, 2013.

### Weight change

Standardized measurements of body weight (to the nearest half kg) were performed by trained staff at the clinical examination at HUNT1 and HUNT2. Relative change in body weight was calculated as percentage change from HUNT1 to HUNT2: 100 x (kg at HUNT2 – kg at HUNT1)/kg at HUNT1. We classified individuals into three categories based on this change score: ‘loss’ (≥5% weight loss), ‘stable’ (< 5% weight change), and ‘gain’ (≥5% weight gain).

### Leisure-time physical activity

At HUNT1, leisure-time physical activity was assessed by the question: ‘How often do you exercise? (By exercise we mean going for walks, skiing, swimming and working out/sports)’, with the response options: ‘never’, ‘less than once a week’, ‘once a week’, ‘2-3 times a week’, or ‘almost every day’. The activity level was dichotomized into ‘inactive’ (‘never’) and ‘active’ (‘less than once a week’, ‘once a week’, ‘2-3 times a week’ or ‘almost every day’).

At HUNT2, leisure-time physical activity was assessed by the question: ‘How much of your leisure-time have you been physically active during the last year? (Think of a weekly average for the year. Your commute to work counts as leisure-time)’. The participants were asked to report number of hours per week of light (no sweating or heavy breathing) and/or hard activity (sweating and heavy breathing) with the response options: ‘none’, ‘<1 hour, ‘1-2 hours’, or ‘≥3 hours’. This information was used to construct a new variable with two categories: ‘inactive’ (‘none’ on both light and hard activity), and ‘active’ (‘<1 h’, ‘1–2 h’ or ‘≥3 h’ of light and/or hard activity). Based on this information, change in physical activity was categorized into four groups combining information from HUNT1 and HUNT2: ‘remained active’, ‘inactive to active’, ‘active to inactive‘, and ‘remained inactive’.

### Other variables

Education was assessed by the question: ‘What is your highest level of education?’, and divided into ‘< 10 years’, ‘10 to 12 years’, and ‘≥13 years’. Smoking status was assessed by questions about past or present use of cigarettes/pipe/cigars, and divided into three categories: ‘never smoked’, ‘former smoker’, and ‘current smoker’. Classification of insomnia was based on the two questions: ‘Have you had problems falling asleep during the last month?’, and ‘During the last month, did you ever wake up too early, not being able to fall asleep again?’ with the following response options: ‘never’, ‘occasionally’, ‘often’, and ‘almost every night’. Participants were classified with insomnia if they answered, ‘often’ or ‘almost every night’ on at least one of the questions. History of cardiovascular disease was assessed by two questions: ‘Have you ever had a myocardial infarction (heart attack)?’ and ‘Have you ever had a stroke/brain haemorrhage?’, with the response options: ‘yes’ and ‘no’. Participants were defined as having a history of cardiovascular disease if they answered ‘yes’ on at least one of the questions. Alcohol consumption was assessed by the question: ‘How many units of beer, wine or spirits do you usually drink in the course of 14 days?’. Participants were then divided into three groups: ‘0 units’, ‘1–4 units’ and ‘≥5 units’. BMI was calculated as weight divided by height squared (kg/m^2^), and classified into one of four groups according to the cut-off points suggested by the World Health Organization [15]: underweight (< 18.5 kg/m^2^), normal weight (18.5–24.9 kg/m^2^), overweight (25.0–29.9 kg/m^2^), or obese (≥30.0 kg/m^2^). Sedentary behavior was assessed by the question: ‘How many hours do you usually spend sitting down during a 24 hour period (work, mealtimes, TV, car, etc.)?’. All potential confounders were assessed at HUNT2, with the exception of BMI, which was assessed at HUNT1.

### Statistical analyses

Cox regression was used to estimate hazard ratios (HR) of the joint association of changes in weight and leisure-time physical activity with all-cause and cardiovascular mortality. The precision of the HR was assessed by 95% confidence intervals (CI). Participants who remained active and had stable weight served as the reference group. All associations were adjusted for age (using age as the time scale in the model). In the multi-adjusted models we adjusted for potential confounding by sex, education (< 10 years, 10 to 12 years, ≥13 years, unknown), smoking (never, former, current smoker, unknown), insomnia (no, yes, unknown), alcohol consumption (0, 1–4 units, ≥5 units, unknown), and history of cardiovascular disease (no, yes, unknown). Potential effect modification between the variables was assessed as departure from additive effects by calculating the relative excess risk due to interaction (RERI) with 95% CI [16].

Sensitivity analyses were conducted to test the robustness of the results. First, the physically active category may include participants who performed very low levels of physical activity. Thus, in order to increase the contrast between inactive and active participants, we excluded participants who reported being physically active less than once a week in HUNT1 and less than one hour a week in HUNT2. Second, as it is likely that the association between weight change and mortality is influenced by initial BMI, we repeated the main analysis adjusting for BMI at HUNT1. Third, since strong evidence shows that sedentary behavior increases the risk of many adverse health conditions [17], we performed sensitivity analyses adjusting for sedentary behavior. Our main analyses were not adjusted for sedentary behavior, as inclusion of different domains of physical behavior may cause collinearity problems in multivariate statistical analyses [18]. Fourth, since the weight loss group was substantially older than the groups of stable weight and weight gain, we also performed an analysis excluding participants < 60 years (we did not have enough statistical power to perform this analysis on participants < 60 years). Fifth, to avoid reverse causation, we performed two sensitivity analyses excluding participants with physical impairments or a history of cardiovascular disease at HUNT2.

Test of Schoenfeld residuals and graphical inspection of log-log plots suggested no important violation of the proportional hazard assumption for the variables included in the regression model. All statistical analyses were performed using Stata for Windows, version 13 (StataCorp LP, College Station, TX, USA).

## Results

During a median follow-up period of 17 years (535,359 person years) a total of 8449 deaths occurred in the cohort. Of these, 3264 died from cardiovascular disease. Characteristics of the study population stratified by weight change categories are presented in Table 1.

**Table 1:** Characteristics of 34,257 participants stratified by change in body weight from HUNT1 to HUNT2

	Change in body weight from HUNT1 to HUNT2		
	Loss (≥5%)	Stable (< 5% change)	Gain (≥5%)
Number of persons, *n*	2701	11,840	19,716
Female, % (*n*)	57 (1546)	43 (5091)	56 (11,063)
Age, mean (SD)	66.7 (14.6)	58.8 (13.6)	51.0 (11.9)
Body Mass Index, mean (SD)	24.6 (4.0)	25.8 (3.7)	27.4 (4.0)
Higher education^a^, % (*n*)	8.5 (229)	14.9 (1769)	18.5 (3642)
Current smokers, % (*n*)	33.3 (898)	28.8 (3407)	25.3 (4992)
Alcohol consumption (≥5 units)^b^, % (*n*)	5.6 (145)	10.2 (1145)	12.4 (2292)
History of cardiovascular disease^c^, % (*n*)	14.0 (380)	7.2 (849)	3.5 (687)
Insomnia symptoms, % (*n*)	17.9 (483)	14.3 (1687)	11.9 (2354)
Weight change in kilograms, mean (SD)	−7.4 (4.0)	0.7 (2.0)	9.0 (4.8)
Remained active	−7.2 (3.8)	0.7 (2.0)	8.9 (4.7)
Inactive to active	−8.1 (5.1)	0.6 (2.1)	9.0 (4.7)
Active to inactive	−7.8 (4.2)	0.5 (2.1)	9.7 (5.4)
Remained inactive	−8.3 (5.2)	0.6 (2.1)	9.9 (5.7)
Physical activity, % (*n*)			
Remained active	74.4 (2009)	84.5 (10,007)	85.6 (16,885)
Inactive to active	8.4 (227)	6.8 (810)	6.8 (1341)
Active to inactive	12.4 (335)	6.3 (746)	5.5 (1092)
Remained inactive	4.8 (130)	2.3 (277)	2.0 (398)

Abbreviations: *SD* standard deviation

^a^College or higher

^b^Over a 2-week period

^c^Myocardial infarction or stroke/cerebral haemorrhage

Table 2 shows the joint association of concurrent changes in weight and leisure-time physical activity with all-cause mortality. Compared to the reference group of participants with stable weight who remained active, participants who remained inactive had a HR for all-cause mortality of 1.54 (95% CI: 1.28–1.85) if they gained weight and 1.29 (95% CI: 1.08–1.53) if they were weight stable, whereas participants who gained weight and remained active had a HR of 1.00 (95% CI: 0.94–1.06). The RERI-estimate for the joint effect of ‘remained active’ versus ‘remained inactive’ among the ‘stable’ versus the ‘gain’ group was 0.26 (95% CI: − 0.13 to 0.66). This suggests a weak synergistic effect of concurrent changes in body weight and physical activity on all-cause mortality. Furthermore, compared to participants with stable weight who remained active, participants who changed their activity level from inactive to active had a HR of 1.22 (95% CI: 1.08–1.38) if they were weight stable and 1.23 (95% CI: 1.09–1.40) if they gained weight. The corresponding HRs for participants who changed their activity level from active to inactive were 1.35 (95% CI: 1.21–1.50) and 1.26 (95% CI: 1.12–1.42), respectively. Weight loss was consistently associated with increased all-cause mortality, irrespective of physical activity changes.

**Table 2:** Combined effect of concurrent changes in weight and leisure-time physical activity on all-cause mortality

Change in leisure -time physical activity	Change in weight^a^								
	Loss (≥5%)			Stable (< 5% change)		Gain (≥5%)			
	Person years/death	Adjusted HR^b^	95% CI	Person years/death	Adjusted HR^b^	95% CI	Person years/death	Adjusted HR^b^	95% CI
All-cause mortality									
Remained active	25,127/1112	1.47	1.37, 1.57	154,446/2879	1.00	Reference	279,598/2204	1.00	0.94, 1.06
Inactive to active	2763/141	1.51	1.27, 1.79	11,934/298	1.22	1.08, 1.38	21,739/257	1.23	1.09, 1.40
Active to inactive	2850/282	1.70	1.50, 1.93	9558/391	1.35	1.21, 1.50	16,577/305	1.26	1.12, 1.42
Remained inactive	1145/106	2.31	1.90, 2.81	3656/132	1.29	1.08, 1.53	5960/122	1.54	1.28, 1.85

Abbreviations: *CI* confidence interval, *HR* hazard ratio

^a^Calculated as change in kilograms between the baseline in HUNT1 and last examination in HUNT2

^b^Adjusted for age, sex, education (< 10 years, 10 to 12 years, and ≥ 13 years, unknown), smoking status (never, former, current, unknown), alcohol consumption (0 units, 1–4 units, ≥5 units, unknown), insomnia symptoms (no, yes, unknown), and history of cardiovascular disease (no, yes, unknown)

Table 3 shows the joint association of concurrent changes in weight and leisure-time physical activity with cardiovascular mortality. Compared to the reference group of participants with stable weight who remained active, participants who remained inactive had a HR for cardiovascular mortality of 1.57 (95% CI: 1.17–2.12) if they gained weight and 1.37 (95% CI: 1.05–1.79) if they were weight stable, whereas participants who gained weight and remained active had a HR of 1.06 (95% CI: 0.96–1.16). The RERI-estimate for these associations was 0.14 (95% CI: − 0.44 to 0.72). Moreover, compared to participants with stable weight who remained active, participants who became active had a HR of 1.32 (95% CI: 1.10–1.59) if they were weight stable and 1.28 (95% CI: 1.04–1.58) if they gained weight, whereas the corresponding HRs for participants who became inactive were 1.60 (95% CI: 1.36–1.86) and 1.46 (95% CI: 1.22–1.75). Weight loss was consistently associated with increased cardiovascular mortality.

**Table 3:** Combined effect of concurrent changes in weight and leisure-time physical activity on cardiovascular mortality

Change in leisure -time physical activity	Change in weight^a^								
	Loss (≥5%)			Stable (< 5% change)		Gain (≥5%)			
	Person years/ death	Adjusted HR^b^	95% CI	Person years/ death	Adjusted HR^b^	95% CI	Person years/ death	Adjusted HR^b^	95% CI
Cardiovascular Mortality									
Remained active	25,127/483	1.56	1.40, 1.74	154,446/1102	1.00	Reference	279,598/802	1.06	0.96, 1.16
Inactive to active	2763/54	1.40	1.06, 1.84	11,934/126	1.32	1.10, 1.59	21,739/96	1.28	1.04, 1.58
Active to inactive	2850/123	1.70	1.41, 2.06	9558/191	1.60	1.36, 1.86	16,577/139	1.46	1.22, 1.75
Remained inactive	1145/44	2.25	1.66, 3.05	3656/58	1.37	1.05, 1.79	5960/146	1.57	1.17, 2.12

Abbreviations: *CI* confidence interval, *HR* hazard ratio

^a^Calculated as change in kilograms between the baseline in HUNT1 and last examination in HUNT2

^b^Adjusted for age, sex, education (< 10 years, 10 to 12 years, and ≥ 13 years, unknown), smoking status (never, former, current, unknown), alcohol consumption (0 units, 1–4 units, ≥5 units, unknown), insomnia symptoms (no, yes, unknown), and history of cardiovascular disease (no, yes, unknown)

### Sensitivity analyses

When excluding participants who reported being physically active less than once a week in HUNT1 and less than one hour a week in HUNT2, the HRs for all-cause and cardiovascular mortality became somewhat stronger (Additional file 1: Table S1), i.e., compared to active persons who had stable weight, inactive participants who gained weight had a HR of 1.60 (95% CI 1.33–1.93) for all-cause mortality and 1.71 (95% CI 1.26–2.31) for cardiovascular mortality. When including BMI at HUNT1 as a covariate in the model, all associations became slightly weaker (Additional file 1: Table S2). Inclusion of sedentary behavior as a covariate in the model did not influence the results. Excluding participants < 60 years had negligible influence on the results for weight loss (Additional file 1: Table S3). Likewise, exclusion of participants with physical impairments had no influences on the results. Finally, exclusion of participants with a history of cardiovascular disease partly attenuated the unfavorable association with cardiovascular mortality in those who gained weight and remained inactive, compared to persons who had stable weight and the same level of physical activity (Additional file 1: Table S4).

## Discussion

This large prospective study showed that the risk of all-cause and cardiovascular mortality is particularly evident among people who gain weight while remaining inactive during a 10–12 year period. Compared to participants with a stable weight who remained physically active during leisure-time, participants who gained weight and remained inactive had a substantially higher all-cause and cardiovascular mortality. In contrast, gaining weight while remaining physically active was not associated with higher mortality. Thus, regardless of maintenance or increase in weight, participants who remained physically active had the lowest risk of premature mortality.

Several prospective studies have shown that leisure-time physical activity may compensate for some of the negative effect of excessive body weight on mortality [8–10]. However, a methodological limitation in these studies is that weight status and physical activity were measured at a single time point, whereas our findings provide evidence of the benefit of long-term maintenance of physical activity, regardless of concurrent weight gain. To our knowledge, this is the first study investigating concurrent long-term changes in body weight and leisure-time physical activity in relation to all-cause and cardiovascular mortality. Lee and colleagues [12] showed that irrespective of change or stability in BMI, men who became less fit had higher all-cause and cardiovascular mortality. However, the latter study investigated change in fitness, which is only partially modifiable due to the genetic determination in human adaptive capacity in response to training [13]. Thus, changes in physical activity may serve as a more attainable goal for initiatives aimed at improving public health.

We found a weak synergistic effect of concurrent changes in weight and physical activity on mortality, underlining the finding that individuals who gain weight while remaining inactive are at a particular risk of premature mortality. Further, it should be noted that individuals who became active had a higher mortality than individuals who remained active, both among those who gained weight and those who were weight stable. However, this risk was still lower than in individuals who remained inactive and gained weight. This may indicate that the benefit of becoming active is not immediate [6], suggesting that the duration of a physically active lifestyle is an important determinant of mortality. Unfortunately, we had no information about when the changes in activity level occurred. Nevertheless, our findings underscore the importance of long-term maintenance of leisure-time physical activity for reducing risk of premature mortality, and suggest that maintenance of an inactive lifestyle is particularly harmful among individuals who gain weight.

With the exception of participants who remained inactive, our results showed that participants who gained weight did not have a higher mortality than stable weight participants with the same level of activity. It is plausible that initial body mass or the duration of excessive body mass may play an important role in the observed association. As weight gain may be less harmful in persons with initially low BMI [19], BMI at HUNT1 may have influenced the observed associations between weight gain, physical activity and mortality. Further, a longitudinal study showed that weight gain earlier in life is associated with higher mortality compared to weight gain later in life [20], whereas Janssen and colleagues [21] demonstrated that the odds of obesity-related comorbidities was increased only among men who had been overweight for ten years or more. However, our results were consistent after adjustment for baseline BMI, suggesting that a weight gain of 5% or more, irrespective of initial BMI, is not detrimental with regards to mortality among individuals who conduct leisure-time physical activity.

In accordance with previous findings [20, 22], our study showed an association between weight loss and higher all-cause and cardiovascular mortality. We found that weight loss was associated with a higher risk of mortality regardless of change in physical activity, as previously found by Østergaard and colleagues [23]. However, our results showed that among individuals who lost weight, individuals who remained inactive had the highest risk of premature mortality. Although our findings suggest that maintenance of physical activity is beneficial in this group, it does not eliminate the increased mortality associated with weight loss. A plausible explanation is reverse causality due to pre-existing or subclinical disease, which may have caused unintentional weight loss [24, 25]. Unfortunately, we had no information about the reason behind the weight loss, nor did we have information about pre-existing disease or physical disabilities.

The strengths of the current study include the large population, which enables analyses of joint categories of changes in weight and physical activity; the prospective design, linking health survey data with data from the Cause of Death Registry that covers all deaths in Norway; and the longitudinal measures of leisure-time physical activity and objectively assessed body weight. We also had detailed information on possible confounders. However, residual confounding by unmeasured or poorly measured factors cannot be ruled out. Another limitation is the assessment of physical activity by questionnaires that is likely subject to measurement error and misclassification. Moreover, physical activity was assessed using different questions at HUNT1 and HUNT2. Although both instruments have shown acceptable validity in terms of objectively assessed measures, the test-retest reliability was higher using HUNT1 [26] than HUNT2 [27] questions. Nevertheless, questionnaire based data are considered adequate to classify people into broad categories, such as inactive/active [28]. This is supported by our sensitivity analysis showing stronger associations with mortality when we increased the contrasts between inactive and active participants. Furthermore, information on possible changes or fluctuations in body weight and physical activity during the relatively long follow-up period was not available. Study findings may also be limited by the restriction to those who survived until HUNT2. Thus, the current study may represent a healthier group in general. Finally, unmeasured or unknown diseases and adverse life events may influence both physical activity and body weight, and thus influence the observed results.

Future studies should examine if changes in physical activity and weight status interact with other factors such as age, sex, and health status. Further, inclusion of objective measures of physical activity may increase knowledge about the effect of different physical behaviors and the impact of activity type and intensity. It should also be noted that recent research indicates a genetic contribution to physical activity [29] and excessive body weight [30, 31]. Increased knowledge about these determinants may therefore improve our understanding about the influence of physical activity and weight gain in the population.

## Conclusions

The current study shows that the risk of all-cause and cardiovascular mortality is particularly evident among people who gain weight while remaining inactive during a 10–12 year period. However, individuals who maintain a physically active lifestyle have the lowest risk of all-cause and cardiovascular mortality, regardless of maintenance or increase in weight. Thus, these findings suggest that there is an interplay between weight gain and leisure-time physical activity that should receive particular attention in the prevention of premature mortality.

## Additional file

Additional file 1: Sensitivity analyses. **Table S1.** Excluded persons who reported being physically active less than once a week in HUNT1 and less than one hour a week in HUNT2. **Table S2.** Included body mass index (BMI) at HUNT1 as a covariate in the model. **Table S3.** Excluded persons aged <60 years. **Table S4.** Excluded persons with a history of cardiovascular disease. (DOCX 23 kb)

## Abbreviations

CI: Confidence interval
HR: Hazard ratio
RERI: Relative excess risk due to interaction
SD: Standard deviation
