# Supplementary material for: Long-term changes in body weight and physical activity in relation to all-cause and cardiovascular mortality: the HUNT study
Source: Int J Behav Nutr Phys Act. 2019 May 20;16:45. doi: 10.1186/s12966-019-0809-2 (PMC6528195; doi:10.1186/s12966-019-0809-2)
Supplement: Supplementary file 1 — Sensitivity analyses. Table S1. Excluded persons who reported being physically active less than once a week in HUNT1 and less than one hour a week in HUNT2. Table S2. Included body mass index (BMI) at HUNT1 as a covariate in the model. Table S3. Excluded persons aged <60 years. Table S4. Excluded persons with a history of cardiovascular disease. (DOCX 23 kb) [file 12966_2019_809_MOESM1_ESM.docx]

**Additional file 1**

**Excluded persons who reported being physically active less than once a week in HUNT1 and less than one hour a week in HUNT2**

Table S1. Combined effect of concurrent changes in weight and leisure-time physical activity on all-cause and cardiovascular mortality.

| Change in leisure time physical activity | Change in weight^a^ | | | | | | | | |
| --- | --- | --- | --- | --- | --- | --- | --- | --- | --- |
|  | Loss (≥5%) | | | Stable (<5% change) | | Gain (≥5%) | | | |
|  | Person years/  death | Adjusted HR^b^ | 95% CI | Person years/  death | Adjusted HR^b^ | 95% CI | Person years/  death | Adjusted HR^b^ | 95% CI |
| All-cause mortality |  |  |  |  |  |  |  |  |  |
| Remained active | 14 548/670 | 1.46 | 1.33, 1.59 | 94 663/1856 | 1.00 | Reference | 159 490/2 204 | 0.99 | 0.92, 1.07 |
| Inactive to active | 2 044/99 | 1.54 | 1.26, 1.88 | 8 474/217 | 1.19 | 1.04, 1.38 | 14 770/257 | 1.22 | 1.04, 1.42 |
| Active to inactive | 1 822/203 | 1.74 | 1.50, 2.02 | 5 169/267 | 1.41 | 1.24, 1.61 | 7 780/305 | 1.36 | 1.17, 1.58 |
| Remained inactive | 1 145/106 | 2.44 | 2.00, 2.98 | 3 656/132 | 1.36 | 1.13, 1.62 | 5 960/122 | 1.60 | 1.33, 1.93 |
| Cardiovascular Mortality |  |  |  |  |  |  |  |  |  |
| Remained active | 14 548/289 | 1.56 | 0.96, 1.91 | 94 663/700 | 1.00 | Reference | 159 490/455 | 1.02 | 0.90, 1.15 |
| Inactive to active | 2 044/35 | 1.36 | 0.96, 1.91 | 8 474/91 | 1.28 | 1.03, 1.60 | 14 770/67 | 1.28 | 1.00, 1.65 |
| Active to inactive | 1 822/290 | 1.82 | 1.45, 2.28 | 5 169/127 | 1.63 | 1.34, 1.98 | 7 780/96 | 1.70 | 1.37, 2.12 |
| Remained inactive | 1 145/44 | 2.46 | 1.80, 3.35 | 3 656/58 | 1.49 | 1.14, 1.95 | 5 960/46 | 1.71 | 1.26, 2.31 |

Abbreviations: CI, confidence interval; HR, hazard ratio

^a^ Calculated as change in kilograms between the baseline in HUNT1 and last examination in HUNT2

^b^ Adjusted for age, sex, education (<10 years, 10 to 12 years, ≥13 years, unknown), smoking status (never, former, current, unknown), alcohol consumption (0 units, 1-4 units, ≥5 units, unknown), insomnia symptoms (no, yes, unknown), and history of cardiovascular disease (no, yes, unknown).

**Included body mass index (BMI) at HUNT1 as a covariate in the model**

Table S2. Combined effect of concurrent changes in weight and leisure-time physical activity on all-cause and cardiovascular mortality.

| Change in leisure time physical activity | Change in weight^a^ | | | | | | | | |
| --- | --- | --- | --- | --- | --- | --- | --- | --- | --- |
|  | Loss (≥5%) | | | Stable (<5% change) | | Gain (≥5%) | | | |
|  | Person years/  death | Adjusted HR^b^ | 95% CI | Person years/  death | Adjusted HR^b^ | 95% CI | Person years/  death | Adjusted HR^b^ | 95% CI |
| All-cause mortality |  |  |  |  |  |  |  |  |  |
| Remained active | 25 127/1 112 | 1.45 | 1.35, 1.55 | 154 446/2 879 | 1.00 | Reference | 279 598/2 204 | 1.01 | 0.95, 1.07 |
| Inactive to active | 2 763/141 | 1.45 | 1.22, 1.71 | 11 934/298 | 1.22 | 1.08, 1.37 | 21 739/257 | 1.23 | 1.08, 1.40 |
| Active to inactive | 2 850/282 | 1.67 | 1.47, 1.89 | 9 558/391 | 1.32 | 1.19, 1.47 | 16 577/305 | 1.25 | 1.11, 1.41 |
| Remained inactive | 1 145/106 | 2.24 | 1.84, 2.73 | 3 656/132 | 1.24 | 1.04, 1.48 | 5 960/122 | 1.51 | 1.26, 1.81 |
| Cardiovascular Mortality |  |  |  |  |  |  |  |  |  |
| Remained active | 25 004/481 | 1.53 | 1.37, 1.70 | 153 431/1 099 | 1.00 | Reference | 275 472/796 | 1.08 | 0.98, 1.19 |
| Inactive to active | 2 744/52 | 1.30 | 0.99, 1.72 | 11 829/126 | 1.33 | 1.10, 1.60 | 21 213/95 | 1.27 | 1.03, 1.57 |
| Active to inactive | 1 132/123 | 1.65 | 1.36, 2.00 | 9 468/189 | 1.54 | 1.32, 1.80 | 16 349/139 | 1.43 | 1.20, 1.71 |
| Remained inactive | 1 132/44 | 2.18 | 1.61, 2.96 | 3 591/58 | 1.30 | 0.99, 1.69 | 5 865/46 | 1.52 | 1.13, 2.05 |

Abbreviations: CI, confidence interval; HR, hazard ratio

^a^ Calculated as change in kilograms between the baseline in HUNT1 and last examination in HUNT2

^b^ Adjusted for age, sex, education (<10 years, 10 to 12 years, ≥13 years, unknown), smoking status (never, former, current, unknown), alcohol consumption (0 units, 1-4 units, ≥5 units, unknown), insomnia symptoms (no, yes, unknown), history of cardiovascular disease (no, yes, unknown) and body mass index at HUNT1 (underweight, normal weight, overweight, obese, unknown).

**Excluded persons aged <60 years**

Table S3. Combined effect of concurrent changes in weight and leisure-time physical activity on all-cause and cardiovascular mortality.

| Change in leisure time physical activity | Change in weight^a^ | | | | | | | | |
| --- | --- | --- | --- | --- | --- | --- | --- | --- | --- |
|  | Loss (≥5%) | | | Stable (<5% change) | | Gain (≥5%) | | | |
|  | Person years/  death | Adjusted HR^b^ | 95% CI | Person years/  death | Adjusted HR^b^ | 95% CI | Person years/  death | Adjusted HR^b^ | 95% CI |
| All-cause mortality |  |  |  |  |  |  |  |  |  |
| Remained active | 25 127/1 112 | 1.47 | 1.36, 1.58 | 154 446/2 879 | 1.00 | Reference | 279 598/2 204 | 1.01 | 0.95, 1.08 |
| Inactive to active | 2 763/141 | 1.57 | 1.32, 1.87 | 11 934/298 | 1.22 | 1.07, 1.39 | 21 739/257 | 1.18 | 1.01, 1.37 |
| Active to inactive | 2 850/282 | 1.71 | 1.51, 1.94 | 9 558/391 | 1.33 | 1.19, 1.49 | 16 577/305 | 1.31 | 1.15, 1.49 |
| Remained inactive | 1 145/106 | 2.33 | 1.90, 2.85 | 3 656/132 | 1.29 | 1.08, 1.55 | 5 960/122 | 1.61 | 1.32, 1.97 |
| Cardiovascular Mortality |  |  |  |  |  |  |  |  |  |
| Remained active | 25 004/481 | 1.54 | 1.38, 1.72 | 153 431/1 099 | 1.00 | Reference | 275 472/796 | 1.05 | 0.95, 1.16 |
| Inactive to active | 2 744/52 | 1.42 | 1.08, 1.88 | 11 829/126 | 1.29 | 1.06, 1.57 | 21 213/95 | 1.25 | 1.00, 1.58 |
| Active to inactive | 1 132/123 | 1.70 | 1.40, 2.06 | 9 468/189 | 1.55 | 1.32, 1.83 | 16 349/139 | 1.46 | 1.21, 1.77 |
| Remained inactive | 1 132/44 | 2.19 | 1.60, 2.99 | 3 591/58 | 1.40 | 1.07, 1.84 | 5 865/46 | 1.63 | 1.19, 2.23 |

Abbreviations: CI, confidence interval; HR, hazard ratio

^a^ Calculated as change in kilograms between the baseline in HUNT1 and last examination in HUNT2

^b^ Adjusted for age, sex, education (<10 years, 10 to 12 years, ≥13 years, unknown), smoking status (never, former, current, unknown), alcohol consumption (0 units, 1-4 units, ≥5 units, unknown), insomnia symptoms (no, yes, unknown), and history of cardiovascular disease (no, yes, unknown).

**Excluded persons with a history of cardiovascular disease**

Table S4. Combined effect of concurrent changes in weight and leisure-time physical activity on all-cause and cardiovascular mortality.

| Change in leisure time physical activity | Change in weight^a^ | | | | | | | | |
| --- | --- | --- | --- | --- | --- | --- | --- | --- | --- |
|  | Loss (≥5%) | | | Stable (<5% change) | | Gain (≥5%) | | | |
|  | Person years/  death | Adjusted HR^b^ | 95% CI | Person years/  death | Adjusted HR^b^ | 95% CI | Person years/  death | Adjusted HR^b^ | 95% CI |
| All-cause mortality |  |  |  |  |  |  |  |  |  |
| Remained active | 22 961/899 | 1.51 | 1.36, 1.58 | 147 142/2 415 | 1.00 | Reference | 272 961/1 917 | 1.01 | 0.95, 1.08 |
| Inactive to active | 2 442/111 | 1.61 | 1.32, 1.87 | 11 049/242 | 1.21 | 1.07, 1.39 | 21 009/225 | 1.31 | 1.01, 1.37 |
| Active to inactive | 2 450/213 | 1.72 | 1.51, 1.94 | 8 888/311 | 1.32 | 1.19, 1.49 | 15 776/245 | 1.30 | 1.15, 1.49 |
| Remained inactive | 1 028/86 | 2.48 | 1.90, 2.85 | 3 388/112 | 1.38 | 1.08, 1.55 | 5 636/96 | 1.54 | 1.32, 1.97 |
| Cardiovascular Mortality |  |  |  |  |  |  |  |  |  |
| Remained active | 25 004/357 | 1.64 | 1.45, 1.86 | 153 431/835 | 1.00 | Reference | 275 472/628 | 1.07 | 0.97, 1.19 |
| Inactive to active | 2 744/39 | 1.58 | 1.14, 2.18 | 11 829/102 | 1.47 | 1.19, 1.81 | 21 213/76 | 1.40 | 1.11, 1.77 |
| Active to inactive | 1 132/85 | 1.81 | 1.44, 2.28 | 9 468/141 | 1.69 | 1.33, 1.91 | 16 349/101 | 1.58 | 1.28, 1.95 |
| Remained inactive | 1 132/33 | 2.50 | 1.76, 3.55 | 3 591/50 | 1.68 | 1.26, 2.24 | 5 865/32 | 1.59 | 1.11, 2.26 |

Abbreviations: CI, confidence interval; HR, hazard ratio

^a^ Calculated as change in kilograms between the baseline in HUNT1 and last examination in HUNT2

^b^ Adjusted for age, sex, education (<10 years, 10 to 12 years, ≥13 years, unknown), smoking status (never, former, current, unknown), alcohol consumption (0 units, 1-4 units, ≥5 units, unknown), insomnia symptoms (no, yes, unknown), and history of cardiovascular disease (no, yes, unknown).
